# Factor XIII Supplementation in Postpartum Hemorrhage: From Biological Rationale to Clinical Implementation

**DOI:** 10.1002/ajh.70181

**Published:** 2025-12-31

**Authors:** Jeremy W. Jacobs, Elizabeth A. Abels, Brian D. Adkins, Garrett S. Booth, Victoria Costa, Sheharyar Raza, Michelle Simon, Jennifer S. Woo, Allison P. Wheeler

**Affiliations:** ^1^ Department of Pathology, Microbiology, and Immunology Vanderbilt University Nashville Tennessee USA; ^2^ Special Coagulation Laboratory Vanderbilt Medical Laboratories Nashville Tennessee USA; ^3^ Department of Obstetrics, Gynecology, and Reproductive Sciences McGovern Medical School Houston Texas USA; ^4^ Division of Transfusion Medicine and Hemostasis, Department of Pathology University of Texas Southwestern Medical Center Dallas Texas USA; ^5^ Department of Pathology NYU Grossman School of Medicine New York New York USA; ^6^ Medical Affairs and Innovation Canadian Blood Services Ottawa Ontario Canada; ^7^ Division of Hematology, Temerty Faculty of Medicine University of Toronto Toronto Ontario Canada; ^8^ Department of Anesthesiology Yale University School of Medicine New Haven Connecticut USA; ^9^ Department of Pathology City of Hope National Medical Center Irvine California USA; ^10^ Division of Pediatric Hematology, Oncology, Bone Marrow Transplant, and Cellular Therapy University of Washington Seattle Washington USA; ^11^ Washington Center for Bleeding Disorders Seattle Washington USA

## Abstract

Postpartum hemorrhage (PPH) remains the leading cause of preventable maternal mortality despite standard interventions. Recent fibrinogen trials failed to improve outcomes, prompting interest in coagulation factor XIII (FXIII). FXIII functions as “molecular cement,” cross‐linking fibrin and stabilizing clots. During pregnancy, FXIII activity decreases 20%–30%, with further depletion during PPH. Observational studies show low antepartum FXIII predicts bleeding risk, while ex vivo supplementation restores clot firmness. The SWIFT trial (NCT06481995) represents the first randomized controlled trial evaluating early FXIII supplementation in PPH. Although implementation challenges are significant (diagnostic accessibility, thrombotic monitoring, supply constraints), even modest hemostatic improvements could substantially reduce maternal mortality.

## 1 Introduction

Postpartum hemorrhage (PPH) remains the leading cause of preventable maternal mortality worldwide, accounting for roughly one quarter of pregnancy‐related deaths [1, 2, 3]. Decades of research and protocol refinement notwithstanding, PPH continues to claim approximately 70 000 maternal lives annually, with rates showing concerning increases in the incidence and severity of PPH in high‐income countries over the past two decades [4]. A further challenge is the lack of a uniform definition. For example, in 2017, the American College of Obstetricians and Gynecologists redefined PPH as cumulative blood loss ≥ 1000 mL or blood loss with signs/symptoms of hypovolemia within 24 h of birth via any route [5, 6]. By contrast, the Royal College of Obstetricians and Gynecologists defines primary PPH as ≥ 500 mL within 24 h of birth (minor 500–1000 mL; major > 1000 mL, often subdivided into moderate 1000–2000 mL and severe ≥ 2000 mL), and secondary PPH as abnormal bleeding 24 h to 12 weeks postpartum [7].

Standard care for PPH denotes guideline‐concordant treatment protocols, which follow a stepwise approach based on clinical assessment of blood loss volume, hemodynamic stability, and ongoing bleeding risk [5, 6]. These protocols are structured sets of interventions delivered together to improve outcomes and include: (i) routine uterotonic prophylaxis for all births (e.g., oxytocin 10 IU as part of active management of the third stage) and additional uterotonics if atony persists; (ii) early tranexamic acid (TXA) for treatment of diagnosed PPH (1 g as soon as PPH is recognized and within 3 h of birth, with a second 1 g if bleeding continues after 30 min or recurs); (iii) stepwise escalation with uterine tamponade/surgical measures and hemostatic support, including massive transfusion protocols and targeted product replacement according to evidence‐based algorithms [3, 5, 6, 7, 8, 9, 10, 11]. Care is multidisciplinary and typically co‐led by obstetrics and anesthesia, in coordination with midwifery, interventional radiology, intensive care, and transfusion medicine.

The persistence of PPH as a leading cause of severe maternal morbidity (SMM) and mortality—even in well‐resourced settings with readily available standard care therapy—underscores both the complexity of obstetric hemostasis and the limitations of current treatment paradigms. Suboptimal implementation of treatment protocols (i.e., the set of time‐critical, evidence‐based actions that should be performed together and consistently for every patient with PPH), such as incomplete or delayed execution and/or low adherence to institutional checklists, contributes to stagnant quality metrics [12]. However, SMM persists even in centers that have successfully optimized their system‐based practices [13]. This therapeutic ceiling is demonstrated by the plateauing of transfusion and hysterectomy rates in high‐income settings and their continued rise in resource‐limited hospitals, emphasizing the need for novel hemostatic targets and therapeutic approaches.

Two of the largest placebo‐controlled trials of early fibrinogen concentrate in PPH showed no reduction in transfusion, blood loss, or other clinical outcomes. In FIB‐PPH, a fixed 2 g dose was administered pre‐emptively at PPH diagnosis (i.e., before fibrinogen results), in addition to standard care [14]. Eligibility included PPH after vaginal delivery with either estimated blood loss (EBL) > 500 mL plus intended manual removal of placenta or EBL > 1000 mL plus intended manual uterine exploration for ongoing bleeding, and after cesarean section with EBL > 1000 mL [14]. In FIDEL, patients with persistent PPH after vaginal delivery requiring escalation from oxytocin to prostaglandins were randomized to 3 g of fibrinogen concentrate or placebo within 30 min of prostaglandin initiation, in addition to guideline‐directed standard care; TXA was permitted after randomization at the investigator's discretion [15].

**FIGURE 1:**
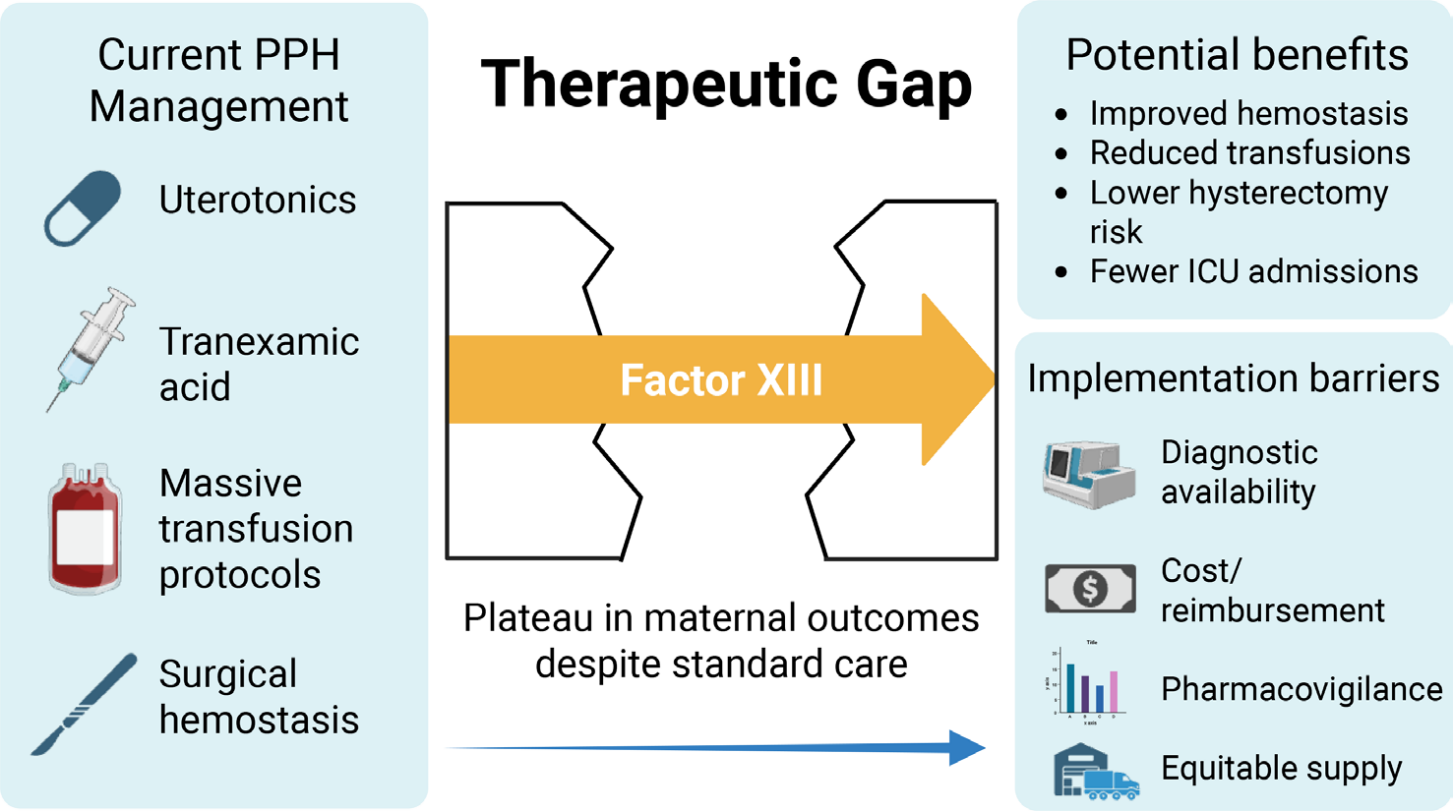
Overview of current postpartum hemorrhage management strategies and how factor XIII replacement may fit into this therapeutic gap. Figure created with BioRender. [Color figure can be viewed at wileyonlinelibrary.com]

Importantly, neither FIB‐PPH nor FIDEL enriched for coagulopathy; baseline fibrinogen was generally within pregnancy ranges, and dosing was not contingent on low fibrinogen. Their neutral results therefore primarily reflect normo‐fibrinogenemic PPH and do not fully address patients with acute obstetric coagulopathy (AOC). AOC is a distinct phenotype characterized by hyperfibrinolysis with acquired dysfibrinogenemia, which may require distinct hemostatic priorities (rapid TXA and restoration of functional fibrinogen) [16]. Nevertheless, the negative or neutral “fibrinogen‐first” trials (Table 1) have prompted exploration of other hemostatic adjuncts with different mechanisms, including coagulation factor XIII (FXIII) (Figure 1). In this critical review, we evaluate FXIII as an adjunct to guideline‐based PPH care from biological rationale to clinical implementation.

**TABLE 1:** Overview of key prior studies of fibrinogen replacement in postpartum hemorrhage, cardiac surgery, and trauma.

Study	Population and timing	Intervention versus comparator	Primary endpoint	Primary finding
Obstetrics				
FIB‐PPH [14]	Early PPH at diagnosis (mostly normofibrinogenemic)	FC 2 g + SOC versus placebo + SOC	RBC transfusion up to 6 weeks postpartum	No reduction in transfusion with pre‐emptive FC
FIDEL [15]	Persistent PPH after vaginal delivery; study drug within 30 min of prostaglandins	FC 3 g versus placebo, both with SOC	Failure of composite of at least 4 g/dL of hemoglobin decrease and/or transfusion of at least two units of RBCs within 48 h following product administration	Administration of 3 g fibrinogen concentrate did not reduce blood loss, transfusion needs or postpartum anemia
OBS2 [17]	Patients with PPH with blood loss of 1000–1500 mL were enrolled. If FIBTEM A5 was ≤ 15 mm and bleeding continued, subjects were randomized to fibrinogen concentrate or placebo	Fibrinogen concentrate (dose titrated by FIBTEM A5, up to 8 g) versus placebo, both with SOC	Number of units of RBCs, plasma, cryoprecipitate and platelets transfused	No improvement in transfusion or bleeding versus placebo
Cardiac surgery				
FIBRES [18]	Post‐CPB bleeding with hypofibrinogenemia	FC 4 g versus cryoprecipitate 10 units	Blood components (RBCs, platelets, plasma) administered during 24 h post CPB	FC is noninferior to cryoprecipitate with regard to number of blood components transfused in a 24‐h period post CPB
REPLACE [19]	Intraoperative, algorithm‐guided first‐line hemostasis	FIBTEM‐guided FC (targeting a FIBTEM MCF of 22 mm) versus placebo/algorithm	Number of units of allogeneic blood products (RBCs, platelets, plasma) administered during the 24 h after medication administration	More allogeneic blood product units were administered and fewer patients avoided transfusion during the first 24 h after FC compared with placebo
ZEPLAST [20]	Postoperative bleeding after complex procedures	FIBTEM MCF guided FC versus placebo	Avoidance of any allogeneic blood product (RBCs, platelets, plasma) during hospital stay up to 30 days	Patients who received FC had a significantly lower rate of any allogeneic blood product transfusion
Bilecen et al. [21]	High‐risk surgery with active intraoperative bleeding	FC versus placebo; dose calculated based on Clauss fibrinogen levels at the end of CPB and dosed to target 2.5 g/L	Intraoperative blood loss measured between intervention (i.e., infusion of study medication after completion of CPB) and closure of the chest when the surgery ended	No significant difference in intraoperative blood loss between FC and control group
Galas et al. [22]	Children with hypofibrinogenemia (plasma fibrinogen < 1 g/L) during cardiac surgery requiring CPB	FC 60 mg/kg or cryoprecipitate 10 mL/kg	Postoperative blood losses during the 48 h after surgery.	The median 48‐h blood loss (intraoperative and 48‐h drainage) was not significantly different between FC and cryoprecipitate
Trauma				
CRYOSTAT‐2 [23]	Injured adults requiring activation of the hospital's major hemorrhage protocol with evidence of active hemorrhage, systolic blood pressure less than 90 mmHg at any time, and receiving at least 1 U of a blood component transfusion	SOC or cryoprecipitate, in which three pools of cryoprecipitate + SOC within 90 min of randomization and 3 h of injury	All‐cause mortality at 28 days	The addition of early and empirical high‐dose cryoprecipitate to SOC did not improve all cause 28‐day mortality
Burt et al. [24]	Patients with traumatic hemorrhage within 4 h of admission to hospital	2025 systematic review and meta‐analysis evaluating the effects of early fibrinogen replacement (cryoprecipitate or FC) versus control	Mortality (28‐day, 30‐day or in‐hospital)	No difference in mortality for FC or cryoprecipitate

Abbreviations: CPB, cardiopulmonary bypass; FC, fibrinogen concentrate; PPH, postpartum hemorrhage; RBC, red blood cell; SOC, standard of care.

## 2 Search Strategy

References for this review were identified through searches of PubMed, the Cochrane Central Register of Controlled Trials, and the Cochrane Database of Systematic Reviews from database inception to July 7, 2025 using the search terms “factor XIII,” “FXIII,” “coagulation factor XIII,” “postpartum hemorrhage” OR “post‐partum hemorrhage,” “obstetric hemorrhage,” and “pregnancy.” Additional references were identified through manual searches of clinical trial registries (ClinicalTrials.gov) and the authors' personal files. Healthcare economic data were obtained from Centers for Medicare & Medicaid Services fee schedules, industry data sheets, and publicly available hospital chargemasters. Laboratory instrumentation and reagent information was sourced from manufacturer websites and product specifications. Only articles published in English were considered. We prioritized human studies involving pregnant or postpartum individuals addressing FXIII activity or supplementation in the context of obstetric bleeding, hemostasis, or coagulopathy. The final reference list was curated to include studies that provided meaningful insights into FXIII biology, clinical applications, diagnostic considerations, and therapeutic potential in obstetric hemorrhage.

## 3 FXIII Biology

Unlike fibrinogen (the substrate for fibrin clot formation) or thrombin (the catalyst), FXIII facilitates the terminal stabilization step of coagulation, the “molecular cement” that cross‐links fibrin. FXIII circulates as a zymogen heterotetramer (FXIII‐A_2_B_2_), with catalytic A subunits and protective B subunits. Thrombin cleavage of the A‐subunit activation peptide, followed by Ca^2+^‐dependent dissociation of the B subunits, produces the active transglutaminase FXIIIa [25, 26].

FXIIIa stabilizes fibrin by forming γ–γ dimers and α‐chain polymers; enhancing RBC retention during clot retraction; and covalently tethering antifibrinolytic proteins to fibrin—principally α_2_‐antiplasmin, with plasminogen activator inhibitor‐2 (PAI‐2) and thrombin‐activatable fibrinolysis inhibitor (TAFI) also reported—thereby increasing clot stiffness and resistance to lysis [27, 28, 29, 30, 31].

FXIII deficiency has clinically important consequences. Reducing FXIII activity substantially accelerates fibrinolysis in vitro—FXIIIa inhibition makes compacted/retracted clots ~8‐fold easier to lyse with tissue‐type plasminogen activator (tPA), while near‐absent FXIII yields clots that dissolve within hours in solubility assays [32]. This instability underlies the characteristic delayed bleeding of severe congenital FXIII deficiency, whereby initial hemostasis may appear intact, followed by secondary hemorrhage as the fibrin scaffold degrades.

Pregnancy places increasing demands on FXIII. Circulating FXIII activity decreases by 20%–30% as gestation progresses, with levels recovering after delivery [33]. This occurs despite rising fibrinogen; increased consumption at the uteroplacental interface and other kinetic changes likely contribute. Clinically, this physiologic fall can unmask or worsen congenital FXIII deficiency, manifesting as recurrent early pregnancy loss, impaired wound healing, and PPH resistant to conventional management.

The clinical relevance of these observations is supported by patient‐level data. In a prospective cohort of 518 patients with objectively measured PPH, FXIII levels in late pregnancy were already below the non‐pregnant reference (median ~54 IU/dL vs. 64–136 IU/dL) and remained ~52 IU/dL at ~1 to 2 L blood loss, while platelet counts remained normal [16]. With progressive hemorrhage, FXIII declined further (median ~27 IU/dL at > 3 L). Outside the acute obstetric coagulopathy group, Clauss fibrinogen stayed within the non‐pregnant reference range across bleed volumes, showing that FXIII levels tracked more closely with hemorrhage severity, though whether FXIII depletion is a mechanistic driver or a sentinel of hemostatic compromise remains under investigation. Nevertheless, FXIII replacement is reasonable to study as an adjunct within guideline‐concordant PPH care (e.g., early TXA) but should not be used as monotherapy.

Beyond PPH, FXIII has been examined in perioperative and trauma settings, with growing interest in its contribution to trauma‐induced coagulopathy (TIC) [34, 35, 36]. In major trauma, acquired FXIII deficiency is present on arrival in approximately 12%–30% of patients (using thresholds of ~60%–70% activity), and often declines further over the first week post injury [36, 37, 38]. Observational studies demonstrate that low FXIII activity (< 60%–70%) in trauma patients is associated with increased bleeding, higher transfusion requirements, impaired clot stability on viscoelastic testing, and worse clinical outcomes, although results have not been uniform across cohorts [37, 38, 39]. European trauma guidelines now suggest monitoring FXIII levels and considering replacement below certain thresholds, though optimal cut‐off values remain debated [40]. In cardiac surgery, a multicenter randomized, placebo‐controlled trial of recombinant FXIII‐A2 restored FXIII activity but did not reduce transfusion or reoperation versus placebo [41, 42]. An earlier small study of FXIII concentrate showed on‐target repletion with acceptable safety [41, 42]. Collectively, these data from the three major clinical settings for acute coagulopathy—trauma, cardiac surgery, and obstetrics—support FXIII as a potentially modifiable contributor to bleeding, though definitive efficacy trials remain limited outside of congenital deficiency.

## 4 FXIII Supplementation

Across two prospective cohorts (*n* = 1848 deliveries), lower pre‐delivery/at‐labor FXIII predicted higher blood loss and PPH [43, 44]. In 548 patients, receiver operating characteristic (ROC) curve cut‐offs were 83.5% for hemorrhage ≥ 500 mL and 75.5% for ≥ 1000 mL [44]. In 1300 patients, each 1% higher FXIII increased the odds of remaining below any blood‐loss threshold (OR 1.011; 95% CI: 1.006–1.015), that is, **~**38.9% higher odds of avoiding PPH ≥ 500 mL with a 30% higher level. Associations persisted after adjustment; fibrinogen showed no detectable association and hemoglobin was non‐significant [43].

Ex vivo and hemodilution models show that supplementing FXIII increases viscoelastic clot firmness (ROTEM MCF) and stability; combined, fibrinogen and FXIII produce larger improvements than FXIII alone, underscoring complementary roles when fibrin(ogen) is adequate [45, 46, 47]. These laboratory findings support the concept that FXIII and fibrinogen address distinct, complementary defects, but clinical outcome data in obstetrics are still needed.

Further, the mechanisms of FXIII and TXA support their complementary potential. TXA blocks lysine‐dependent binding of plasminogen (and plasmin) to fibrin, thereby suppressing plasmin generation and fibrinolysis with little effect on clot firmness or thrombin generation [46, 48, 49, 50, 51]. FXIIIa stabilizes fibrin, which increases viscoelastic clot firmness (increased MCF; sometimes decreased CFT) with smaller, context‐dependent effects on lysis—less than TXA [46, 49, 50, 51]. In RBC‐containing clots, FXIII strengthens the fibrin network, promotes RBC retention, and augments TXA's antifibrinolytic effect [29]. Taken together, TXA targets fibrinolysis, whereas FXIII may help when FXIII activity is low or clot stability is poor [32]. Head‐to‐head/combination trials in PPH are lacking; a definitive study would randomize licensed FXIII concentrate vs. placebo on top of standardized early TXA and guideline care, with mechanistic sub‐studies (FXIII activity; ROTEM/TEG firmness and lysis) and clinical endpoints.

The converging biological, clinical, and mechanistic considerations led to the initiation of SWIFT (NCT06481995) in July 2024, with an estimated completion date of December 2028 (Table 2) [52, 53]. This multicenter, randomized study tests whether early FXIII replacement, added to standard care, reduces measured postpartum blood loss within 24 h versus standard care alone [52, 53]. This trial spans nine Swiss centers; all participants receive 1 g TXA at measured blood loss (MBL) ≥ 500 mL. Randomization occurs when bleeding continues and exceeds 700 mL. The intervention group receives plasma‐derived FXIII (pdFXIII) concentrate (Fibrogammin) as a single fixed dose based on body weight (1250 IU if < 80 kg; 1500 IU if 80–99.9 kg) in addition to obstetric standard care; controls receive standard care alone. FXIII dosing is not titrated using FXIII activity levels, but FXIII activity is measured for safety monitoring purposes. SWIFT does not mandate a specific FXIII assay, but sites use their local, validated FXIII activity result to identify the prespecified safety‐net threshold (< 60%), which may trigger protocol‐defined escalation of care. Beyond the primary endpoint of reduced blood loss, secondary outcomes include transfusion, major interventions, and safety.

**TABLE 2:** SWIFT trial in brief.

Item	Summary
Registry/acronym	NCT06481995 (“SWIFT”)
Design	Multicenter, randomized (1:1), open‐label, add‐on to standard care
When randomized	After delivery once measured blood loss ≥ 700 mL with ongoing bleeding; TXA 1 g IV given at 500 mL per protocol
Intervention	Plasma‐derived FXIII concentrate single IV dose (1250 IU if < 80 kg or 1500 IU if 80–99.9 kg)
Comparator	Standard care only (no placebo)
Standard care window	Guideline‐concordant PPH treatment protocol; restrictions on additional coagulation factors/repeat TXA until prespecified MBL thresholds or safety‐net labs trigger escalation
Primary outcome	Measured blood loss within 24 h (standardized QBL)
Key secondary outcomes	Transfusion; MBL ≥ 2000 mL; procedures (embolization, uterine‐sparing sutures/ligation, hysterectomy); ICU; safety including thromboembolic events; labs to ~48 h
Follow‐up	Maternal outcomes and safety to ~6 to 9 weeks postpartum
Notes	Mechanistic sub‐studies at selected centers (serial factor measurements)

*Note:* The complete protocol is open access [52].

Abbreviations: ICU, intensive care unit; IV, intravenous; MBL, measured blood loss; PPH, postpartum hemorrhage; TXA, tranexamic acid; QBL, quantitative blood loss.

## 5 Diagnostics: Measuring FXIII Activity During PPH

The integration of FXIII into clinical practice depends on timely, accurate diagnostic testing. Unlike fibrinogen, which can be evaluated in routine coagulation laboratories and estimated through rapid assays, FXIII assessment requires specialized capabilities that vary significantly in their speed, accuracy, and clinical utility.

### 5.1 Historical Methods and Their Limitations

Qualitative clot‐solubility tests were the historical screen for severe FXIII deficiency. In these assays, patient plasma is clotted with thrombin and calcium, then exposed to chaotropic agents (5M urea or 1% monochloroacetic acid), which disrupt non‐covalent fibrin interactions while covalent FXIII‐mediated cross‐links resist dissolution. Complete clot dissolution indicates severe FXIII deficiency; persistence suggests adequate cross‐linking. These tests are technically simple and inexpensive but detect only extreme depletion (< 2–10 IU/dL), require assessment at 24 h, and can yield false‐positives when fibrinogen is low. Contemporary guidelines now advise against their routine use [54].

### 5.2 Contemporary Quantitative Methods

Photometric ammonia‐release assays are the predominant methodology for clinical FXIII quantitation. These automated assays exploit FXIIIa transglutaminase activity—after thrombin/Ca^2+^ activation, FXIIIa catalyzes acyl transfer between a glutamine donor and an amine acceptor, releasing ammonia; a coupled glutamate‐dehydrogenase reaction consumes NH_3_, and the drop in NAD(P)H at 340 nm is proportional to FXIII activity [55].

Modern platforms can generate results within 10–15 min of sample processing, theoretically yielding total laboratory turnaround times of 15–20 min—potentially fast enough to guide real‐time therapeutic decisions during active PPH. The assays feature broad analytical ranges (5–200 IU/dL) with excellent precision, though technical considerations include background NAD(P)H oxidation necessitating appropriate plasma blanks, as well as interference from hemolysis and lipemia.

### 5.3 Emerging Technologies

Fluorogenic isopeptidase assays represent a newer approach that exploits the reciprocal FXIIIa‐catalyzed reaction (cleavage of γ–ε isopeptide bonds) to generate fluorescent signals directly proportional to enzyme activity [55]. These assays offer superior analytical sensitivity (detection limits ~0.8 IU/dL) and eliminate the need for plasma blanks, but remain research‐use‐only in some jurisdictions, and some researchers question whether isopeptidase activity appropriately mirrors fibrin cross‐linking biology.

### 5.4 Non‐Activity Assays

Immunologic antigen assays quantify FXIII protein mass rather than activity, useful for confirmatory work‐up of congenital deficiency. Turn‐around is assay‐platform dependent—90–120 min for enzyme‐linked immunosorbent assays (ELISA), 15 min for on‐board latex methods—but most testing is performed at reference laboratories, yielding multi‐day turnaround times.

### 5.5 Point‐of‐Care Assays

Viscoelastic hemostatic assays (TEG, ROTEM, Quantra) profile clot formation, strength, and fibrinolysis, and suggest impaired fibrin cross‐linking and may be used pragmatically to guide care. After fibrinogen repletion (and TXA if given), a persistently low or fibrin‐based firmness parameter and/or increased lysis can be *compatible* with FXIII depletion, prompting FXIII testing or empiric replacement [56, 57]. However, at present, this is a theoretical, expert‐opinion approach—these signals are indirect and non‐specific, as FIBTEM MCF is driven primarily by fibrinogen, with FXIII providing an additional (albeit smaller) contribution to clot firmness and stability, and is notably demonstrable when FXIII is very low or after in vitro FXIII supplementation [58]. Further, because obstetric thresholds for diagnosing FXIII deficiency are unvalidated, FXIII activity remains the standard; accordingly, viscoelastic assays were not mandated for eligibility or dosing in the SWIFT protocol.

## 6 Implementation Challenges

The translation of FXIII research into clinical practice faces several significant challenges that must be addressed to ensure safe and effective implementation.

### 6.1 Laboratory Infrastructure and Diagnostic Accessibility

The most immediate barrier to FXIII implementation may be diagnostic rather than therapeutic. Due to the specialized testing requirements for quantitative FXIII activity, many facilities rely on send‐out testing with impractical delays for hyperacute PPH scenarios. This diagnostic gap creates a fundamental implementation paradox: clinicians cannot safely use FXIII supplementation without reliable monitoring, but laboratories cannot justify implementing FXIII testing without demonstrated clinical demand. This may necessitate initial empiric dosing protocols guided by clinical severity and viscoelastic testing until FXIII assays become widely available.

Economic barriers compound this challenge. While reagent costs are modest [59], reimbursement rates are low [60] and hospital chargemasters show wide variability [61]. Together, low reimbursement and variable charges can disincentivize onsite testing and indirectly promote empiric treatment pathways, potentially resulting in under‐ or over‐use. Coordinated efforts between obstetric services, laboratory directors, and diagnostic manufacturers will be essential to prioritize FXIII testing infrastructure in hospitals managing high‐risk deliveries.

### 6.2 Thrombotic Risk and Safety Monitoring

The primary safety concern with FXIII supplementation, and thus the need for clinical monitoring of FXIII activity, is the hypothetical potential for thrombotic complications. Because FXIII enhances clot stability and resistance to fibrinolysis, excessive supplementation could theoretically tip the hemostatic balance toward pathological thrombosis. This risk is particularly relevant in pregnancy, where baseline thrombotic risk is already elevated 4–5 fold compared to non‐pregnant individuals [62]. The ongoing SWIFT trial partially addresses this concern by including thromboembolic events and other maternal adverse outcomes among safety endpoints and excluding patients with prior venous thromboembolism; however, routine clinical practice cannot perfectly impose such exclusion criteria.

Long‐term pharmacovigilance suggests low reported rates of thrombosis with pdFXIII. In CSL Behring data (June 1993–September 2013), 1.65 billion IU were distributed (1 181 036 “standard 70 kg doses”), with seven reports classified as possible thromboembolic events—approximately 1 per 236 207 200 IU or 1 per 168 700 doses [63]. Spontaneous reports in FAERS also include thrombotic events with FXIII concentrate, but such data lack denominators and may include duplicates/confounding and therefore cannot be used to estimate incidence. Extrapolation to acute PPH remains uncertain; ongoing pharmacovigilance (ideally linking maternity registries to product utilization) and FXIII trials should prespecify venous/arterial thrombosis and organ‐dysfunction outcomes.

### 6.3 Supply Chain and Global Equity

Two licensed FXIII concentrates are currently available, pdFXIII (Fibrogammin/Corifact) and recombinant FXIII (catridecacog; Tretten/NovoThirteen), which only contains FXIII‐A_2_. After reconstitution, pdFXIII contains ~62.5 IU/mL FXIII, while rFXIII has ~833 IU/mL of FXIII; neither contains clinically meaningful amounts of fibrinogen (Table 3) [64]. Alternative sources of FXIII, which do contain fibrinogen, include fibrinogen concentrates such as Fibryga, which provides 1 g fibrinogen per 50 mL (~20 mg/mL) and—per independent characterization—retains 0.20 IU of FXIII per mg of fibrinogen (~200 IU FXIII per 1 g vial), resulting in a final FXIII concentration of ~4 IU/mL after reconstitution [65]. Cryoprecipitate also provides fibrinogen and FXIII, with a similar FXIII/fibrinogen ratio of approximately 0.30 IU FXIII per mg of fibrinogen (FXIII: ~60 ± 30 IU per unit; volume ~15–25 mL) and typical fibrinogen content ~150–300 mg/unit, yielding FXIII concentration of ~2 to 4 IU/mL and fibrinogen ~8 to 12 mg/mL [66].

**TABLE 3:** Approximate FXIII content and concentration by product.

Product	FXIII per vial/unit (approximate)	Fibrinogen per vial/unit (approximate)	FXIII concentration per example dose (approximate)	Fibrinogen concentration per example dose (approximate)	Fibrinogen (mg):FXIII (IU)	Notes/source
rFXIII (catridecacog; NovoThirteen/Tretten)	2500 IU per 3 mL vial	0	833 IU/mL (standard dose is 35 IU/kg)	0	N/A	rFXIII only contains the FXIII A‐subunit. https://www.ema.europa.eu/en/documents/product‐information/novothirteen‐epar‐product‐information_en.pdf
pdFXIII (Fibrogammin/Corifact)	250 IU (4 mL) or 1250 IU (20 mL)	0	~62.5 IU/mL (standard dose is 35 IU/kg)	0	N/A	Plasma‐derived FXIII https://www.medicines.org.uk/emc/product/3736/smpc?
Fibrinogen concentrate (Fibryga)	~200 IU per 1 g vial (50 mL)	1 g per 50 mL vial	~4 IU/mL (~800 IU/200 mL)	~20 mg/mL (4 g/200 mL)	~5:1	Regulatory labeling for Fibryga specifies 1 g/50 mL (20 mg/mL) fibrinogen but does not report FXIII content; independent characterization measured ~0.20 IU FXIII per mg fibrinogen (~4 IU/mL after reconstitution). Schulz PM, Gehringer W, Nöhring S, et al. Biochemical characterization, stability, and pathogen safety of a new fibrinogen concentrate (fibryga). *Biologicals*. 2018;52:72–77. doi:10.1016/j.biologicals.2017.12.003
Fibrinogen concentrate (RiaSTAP)	~55 IU per 1 g vial (50 mL)	900–1300 mg per vial (50 mL)	~1.1 IU/mL (~220 IU/200 mL)	~20 mg/mL, range: 18–26 mg/mL (4/200 mL, range: 3.6–5.2 g/200 mL)	~18.2:1	Regulatory labeling for RiaSTAP specifies reconstitution to ~20 mg/mL fibrinogen (900–1300 mg/vial in 50 mL) and does not report FXIII content. Independent studies demonstrate low co‐purified FXIII. Stanford S, Roy A, Cecil T, et al. Differences in coagulation‐relevant parameters: Comparing cryoprecipitate and a human fibrinogen concentrate. *PLoS One*. 2023;18(8):e0290571. doi:10.1371/journal.pone.0290571
Cryoprecipitate (single unit)	~60 ± 30 IU per unit (~15 to 25 mL)	150–300 mg/unit (~15 to 25 mL)	~2 to 4 IU/mL (~600 IU per standard dose = 10 units, ~150 to 250 mL)	~8 to 12 mg/mL (~1.5 to 3.0 g per standard dose = 10 units, ~150 to 300 mL)	~3:1	Regulatory labeling for cryoprecipitate identifies FXIII as a component but does not quantify it; measured content is ~60 ± 30 IU per unit (mean 21.3 mL; ~2.8 IU/mL), with substantial lot‐to‐lot variability. Caudill JS, Nichols WL, Plumhoff EA, et al. Comparison of coagulation factor XIII content and concentration in cryoprecipitate and fresh‐frozen plasma. *Transfusion*. 2009;49(4):765–770. doi:10.1111/j.1537‐2995.2008.02021.x

*Note:* “Standard dose” shown for fibrinogen products is 4 g; for cryo, 10 units. FXIII‐only products (rFXIII, pdFXIII) do not contain fibrinogen; their “per standard dose” FXIII concentration varies with patient weight/IU dose.

Abbreviations: IU, international units; pdFXIII, plasma‐derived factor XIII concentrate; rFXIII, recombinant factor XIII concentrate.

When comparing standard adult repletion doses, 4 g Fibryga delivers ~4 g fibrinogen and ~800 IU FXIII (200 mL total volume), whereas 10 units of cryoprecipitate deliver ~1.5 to 3.0 g fibrinogen and ~600 IU FXIII (150–250 mL total volume). By comparison, a dose of pdFXIII concentrate delivers either 250 IU (4 mL) or 1250 IU (20 mL), and recombinant FXIII provides 2500 IU (3 mL).

The availability and accessibility of these different FXIII sources varies dramatically across healthcare systems, creating potential disparities in treatment options. In high‐income countries, the choice between dedicated FXIII concentrates, cryoprecipitate, and FXIII‐containing fibrinogen concentrates may be dictated by formulary decisions, cost considerations, and institutional preferences. For instance, hospitals may favor cryoprecipitate for dual fibrinogen‐FXIII replacement despite its larger volume requirements, while others may prefer the precision and reduced volume of dedicated concentrates. Financial costs likely vary widely by product type, dose, and region, but generally exceed other routine hemostatic agents [64, 67]. However, if FXIII concentrate is shown to be effective, costs must be weighed against potential savings from reduced transfusion requirements, shorter hospital stays, and avoidance of major surgical interventions.

In resource‐limited settings, these cost differentials become prohibitive barriers to access. Many low‐ and middle‐income countries lack reliable access to any plasma‐derived concentrates, relying instead on whole blood or plasma for hemostatic support [68, 69]. Even cryoprecipitate, while theoretically more affordable, requires specialized blood banking infrastructure for production and storage that may not be available in settings where PPH mortality is highest. This creates a scenario where the most expensive and sophisticated FXIII products are available primarily in countries with the lowest maternal mortality rates, while the highest‐burden regions have limited access to any form of factor replacement.

Current FXIII concentrate supply reflects use in an ultra‐rare congenital disorder. Between 1993 and 2013, approximately 1.65 billion IU of pdFXIII were distributed globally (~82.5 million IU/year) [55]. At labeled prophylaxis (~40 IU/kg every 28 days; ~36 400 IU/year for a 70 kg adult), this supports ~2300 “70‐kg patient‐years” annually—consistent with an ultra‐rare global prevalence (~1 in 1–3 million). If obstetric indications were adopted widely, even modest uptake (e.g., tens of thousands of PPH doses annually) could strain supply, underscoring the need for coordinated forecasting to protect access for patients with congenital deficiency.

## 7 Future Directions and Research Priorities

The path forward for FXIII in PPH management requires a coordinated research agenda addressing both clinical efficacy and implementation challenges.

### 7.1 Clinical Research Priorities

Beyond the SWIFT trial, several key research questions require urgent attention. First, optimal dosing strategies remain undefined—should FXIII be dosed based on body weight, baseline FXIII activity, or bleeding severity? Second, the timing of administration requires clarification—is prophylactic supplementation in high‐risk patients superior to reactive treatment after bleeding begins? Third, combination strategies with TXA and other hemostatic agents need systematic evaluation to identify complementary regimens.

### 7.2 Implementation Challenges

Successful translation of FXIII research into practice will require robust implementation studies addressing workflow integration, provider education, and system‐level barriers. Key questions include: How can FXIII testing be integrated into existing massive transfusion protocols? What training is required for obstetric and anesthesia teams? How can supply chain management ensure consistent availability without waste?

### 7.3 Global Health Applications

The potential for FXIII supplementation to reduce maternal mortality in resource‐limited settings deserves special attention. Novel delivery strategies, including point‐of‐care testing algorithms and simplified dosing protocols, could make this intervention accessible in settings where sophisticated laboratory support is unavailable. Partnerships with coagulation assay manufacturers, global health organizations, and pharmaceutical companies will be essential to ensure utility and equitable access.

## 8 Conclusion

Fibrinogen‐first strategies have not improved outcomes in unselected PPH, renewing interest in FXIII, a physiologic stabilizer of fibrin that declines during obstetric bleeding and is replaceable with licensed concentrates. SWIFT will test whether early FXIII, added to guideline‐based care including TXA, reduces blood loss and transfusion. If efficacy is shown, implementation will hinge on rapid FXIII testing, thrombotic surveillance, reliable supply, and protocol integration; even modest hemostatic gains could reduce maternal morbidity and mortality. Obstetric programs should begin readiness planning—laboratory capacity for FXIII activity assays, safety monitoring frameworks, and procurement forecasting—to enable safe, equitable uptake if the evidence supports it.

## Author Contributions

J.W.J. performed the literature search, collected and analyzed the data, and wrote the first draft. G.S.B. and S.R. searched the literature, collected and analyzed the data, and revised the manuscript. A.P.W. analyzed the data and revised the manuscript. E.A.A., B.D.A., V.C., M.S., and J.S.W. revised the manuscript. All authors approved the final version.

## Funding

The authors have nothing to report.

## Ethics Statement

This article is a review based on previously published studies and publicly available data. No new studies involving human participants or animals were conducted by the authors for this work; therefore, institutional review board/ethics committee approval and informed consent were not required.

## Conflicts of Interest

None of the authors have disclosures related to this article. Unrelated, J.W.J. reports research funding from Bayer and honorarium from Instrumentation Laboratories. A.P.W. reports having received an honorarium for advisory board participation from Sanofi, Novo Nordisk, Ceres, Hemab Therapeutics, Pfizer, Genentech, and Takeda.

## Data Availability

Data sharing not applicable to this article as no datasets were generated or analysed during the current study.
